# Low-Intensity and Short-Duration Continuous Cervical Transcutaneous Spinal Cord Stimulation Intervention Does Not Prime the Corticospinal and Spinal Reflex Pathways in Able-Bodied Subjects

**DOI:** 10.3390/jcm10163633

**Published:** 2021-08-17

**Authors:** Atsushi Sasaki, Roberto M. de Freitas, Dimitry G. Sayenko, Yohei Masugi, Taishin Nomura, Kimitaka Nakazawa, Matija Milosevic

**Affiliations:** 1 Department of Life Sciences, Graduate School of Arts and Sciences, The University of Tokyo, Tokyo 153-8902, Japan; atsushi-sasaki@g.ecc.u-tokyo.ac.jp (A.S.); ymasugi@tiu.ac.jp (Y.M.); nakazawa@idaten.c.u-tokyo.ac.jp (K.N.); 2 Japan Society for the Promotion of Science, Tokyo 102-0083, Japan; 3 Department of Mechanical Science and Bioengineering, Graduate School of Engineering Science, Osaka University, Osaka 560-8531, Japan; r.freitas@bpe.es.osaka-u.ac.jp (R.M.d.F.); taishin@bpe.es.osaka-u.ac.jp (T.N.); 4 Center for Neuroregeneration, Department of Neurosurgery, Houston Methodist Research Institute, Houston, TX 77030, USA; dgsayenko@houstonmethodist.org; 5 School of Health Sciences, Tokyo International University, Saitama 350-1197, Japan

**Keywords:** cervical, transcutaneous spinal cord stimulation, corticospinal pathway, spinal reflex, neuromodulation

## Abstract

Cervical transcutaneous spinal cord stimulation (tSCS) has been utilized in applications for improving upper-limb sensory and motor function in patients with spinal cord injury. Although therapeutic effects of continuous cervical tSCS interventions have been reported, neurophysiological mechanisms remain largely unexplored. Specifically, it is not clear whether sub-threshold intensity and 10-min duration continuous cervical tSCS intervention can affect the central nervous system excitability. Therefore, the purpose of this study was to investigate effects of sub-motor-threshold 10-min continuous cervical tSCS applied at rest on the corticospinal and spinal reflex circuit in ten able-bodied individuals. Neurophysiological assessments were conducted to investigate (1) corticospinal excitability via transcranial magnetic stimulation applied on the primary motor cortex to evoke motor-evoked potentials (MEPs) and (2) spinal reflex excitability via single-pulse tSCS applied at the cervical level to evoke posterior root muscle (PRM) reflexes. Measurements were recorded from multiple upper-limb muscles before, during, and after the intervention. Our results showed that low-intensity and short-duration continuous cervical tSCS intervention applied at rest did not significantly affect corticospinal and spinal reflex excitability. The stimulation duration and/or intensity, as well as other stimulating parameters selection, may therefore be critical for inducing neuromodulatory effects during cervical tSCS.

## 1. Introduction

Spinal cord stimulation (SCS) has been applied in rehabilitation as a neuromodulation application to assist recovery of sensory and motor function after spinal cord injury (SCI), with remarkable achievements in clinical research [1]. Specifically, it has been demonstrated that lumbar epidural stimulation can contribute to restoration of voluntary motor control of lower-limb muscles, postural stability, and gait function in chronic SCI individuals [2,3,4]. Similarly, cervical epidural SCS has been used for promoting volitional hand function in individuals with chronic tetraplegia [5]. Consistent with epidural SCS approaches, non-invasive transcutaneous spinal cord stimulation (tSCS) has also been utilized successfully in applications for improving sensory and motor function during lower-limb voluntary movement [6] and walking using lumbar stimulation [7,8,9,10,11,12], trunk stability and standing with lower thoracic and lumbar stimulation [13,14], and gripping and upper-limb function with cervical stimulation [15,16,17,18,19]. Computer simulations and experimental studies using animal models as well as human studies have shown compelling evidence that electric impulses induced by either implanted epidural or surface non-invasive electrodes can primarily activate the afferent fibers in the posterior roots of the spinal cord [20,21,22,23,24,25]. Since the stimulated afferent fibers activated by tSCS have synaptic connections to the spinal interneurons and motoneurons, tSCS application can therefore be utilized to modulate spinal motor excitability.

Due to the importance of walking in humans, lumbar tSCS applications for modulation of spinal locomotor circuits have been the main focus of many previous studies [7,8,9,10,11,12]. Although cervical tSCS is also expected to have similar neuromodulatory effects on the upper-limb muscle function, it has been unexplored relative to lumbar tSCS application until recently. Recent reports have shown impressive therapeutic effects of continuous cervical tSCS [16,17,18,26]. However, little is known about the neural mechanisms and how continuous cervical tSCS affects central nervous system (CNS) excitability and the innervated upper-limb muscles.

Several recent studies have reported effects of continuous cervical tSCS intervention on neural excitability of upper-limb muscles [15,19]. Specifically, Benavides et al. [15] demonstrated that upper-limb subcortical but not cortical motor-evoked potentials, and intracortical inhibition can be facilitated after 20 min of continuous cervical tSCS, suggesting that cervical tSCS has an excitatory effect on the spinal networks and an inhibitory effect on the cortical networks. Moreover, it was shown that these effects were similar for able-bodied and SCI patients [15], indicating that studies with able-bodied participants can also be translated for rehabilitation protocols in SCI patients. Similarly, another study also demonstrated that continuous cervical tSCS applied intermittently (20 s ON/80 s OFF) at rest in able-bodied participants for 30 min can affect the F-wave amplitude, which indicates the excitability of anterior horn motoneurons in the spinal cord [19].

Taken together, these studies revealed that relatively long (20–30 min), continuous cervical tSCS interventions can modulate spinal and/or cortical networks controlling the upper-limb muscles after the intervention duration. However, the effect of a single, short intervention (<10 min) and understanding of how neural activity is modulated by the intervention remain unclear. Shortening the intervention duration and using low-intensity stimulation may improve therapy adherence for patients in an effort to minimize discomforts in actual clinical therapy since it was reported that high-intensity cervical tSCS could induce discomfort [27].

Therefore, the aim of this study was to investigate how low-intensity and short-duration continuous cervical tSCS applied at rest could affect corticospinal and spinal reflex excitability before, during, and after the intervention. We hypothesized that short-term application of low-intensity continuous tSCS would affect corticospinal and spinal circuits of upper-limb muscles [10,15,19,28]. To test our hypotheses, we used transcranial magnetic stimulation (TMS) to investigate motor-evoked potentials (MEPs), which reflect corticospinal excitability [29]. Moreover, single-pulse cervical tSCS was used to investigate spinal reflex responses, which reflect the excitability of the spinal reflex circuits in multiple upper-limb muscles.

## 2. Materials and Methods

### 2.1. Participants

Ten able-bodied individuals were recruited in our current study (25.8 ± 2.6 years, 68.8 ± 8.3 kg, and 173.4 ± 5.1 cm (mean ± SD)). None of the participants had a history of neurological and musculoskeletal impairments. All participants gave written informed consent in accordance with the Declaration of Helsinki. The experimental procedures were approved by the local institutional ethics committee at the Graduate School of Arts and Sciences at The University of Tokyo.

### 2.2. Experimental Procedures

**Figure 1:**
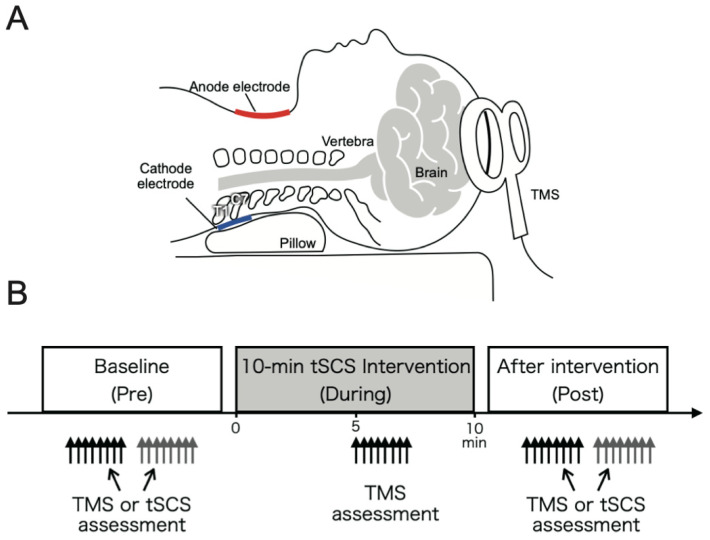
(**A**) Experimental setup showing head posture of participants during the experiments, including the cervical transcutaneous spinal cord stimulation (tSCS) setup and transcranial magnetic stimulation (TMS) setup. (**B**) TMS assessments were conducted before (Pre), during continuous cervical tSCS (During), and after (Post) the intervention, while tSCS assessments were conducted at Pre and Post intervention time points. At each assessment point, TMS or tSCS stimuli consisted of 8 elicited responses. In the Pre and Post assessment, the order of TMS and tSCS assessments was randomized between participants.

During the experiments, participants remained in the supine position (Figure 1A). Assessments consisted of evaluating corticospinal excitability through motor-evoked potential (MEP) elicited by single-pulse TMS (see Section 2.3.2) and spinal reflex circuits excitability through posterior root muscle (PRM) reflexes elicited by single-pulse cervical tSCS (see Section 2.3.3). Assessments were performed (i) at baseline before the intervention (Pre); (ii) at mid-point during the intervention (5 min after the start of the intervention; Figure 1B) (During); and (iii) immediately after the end of the 10-min intervention (Post). MEP responses were assessed in Pre, During, and Post, while PRM reflex responses were assessed in Pre and Post since same electrodes were used for eliciting PRM reflex responses and for delivering the intervention. Eight MEP (TMS) and PRM reflex (tSCS) responses were elicited in each assessment phase. Both assessments were performed in the same session, while the order of TMS or tSCS assessments was pseudorandomized between participants (Figure 1B).

### 2.3. Assessments

#### 2.3.1. Electromyography (EMG) Activity

Electromyographic (EMG) activity was recorded unilaterally from the right (dominant in all the subjects) hand: (1) biceps brachii (BB), (2) triceps brachii (TB), (3) flexor carpi radialis (FCR), (4) extensor carpi radialis (ECR), (5) first dorsal interosseous (FDI), and (6) abductor pollicis brevis (APB) muscles. Surface EMG electrodes (Ag/AgCl; Vitrode F-150S; Nihon Kohden, Tokyo, Japan) were placed on the muscle belly of the right upper limb with an approximate interelectrode distance of 20 mm, whereas APB electrodes were placed over the muscle belly and first metacarpophalangeal joint, and FDI electrodes were placed over the muscle belly and the second metacarpophalangeal joint [23]. A reference electrode was placed around the lateral epicondyle. Prior to application of electrodes, skin was cleaned using alcohol swabs to reduce skin impedance. Signals were band-pass filtered (5–1000 Hz) and amplified (×1000) using a multi-channel EMG amplifier (MEG-6108, Nihon Kohden, Tokyo, Japan). All data were digitized at a sampling frequency of 4000 Hz using an analog-to-digital converter (PowerLab/16SP, AD Instruments, Castle Hill, Australia) and stored on a computer for post processing.

#### 2.3.2. Transcranial Magnetic Stimulation (TMS)

TMS was delivered over the primary motor cortex using a mono-phasic magnetic stimulator (Magstim 200, Magstim Co., Whitland, UK) through a figure-of-eight coil (Magstim Co., Whitland, UK). The optimal stimulation spot (“hot spot”) was searched over the left cortex, where MEPs could be recorded from the right FDI muscle. The motor threshold (MT) was determined while the participants remained in the supine position and relaxed. MT was defined as the minimum TMS intensity for which FDI MEPs had a peak-to-peak amplitudes larger than 50 μV and were evoked in at least five out of ten successive trials [30]. The stimulation intensity for experiment was set at 120% of the MT level (1.2MT) (62.3 ± 11.3 % of maximal stimulator output). MEPs were simultaneously recorded from the FDI, APB, FCR, and ECR muscles, although stimulation “hot spot” was optimized for the FDI muscle. Specifically, the mean amplitudes of the MEPs of the FDI, APB, FCR, and ECR muscles during Pre phase were 0.12 ± 0.13, 0.16 ± 0.12, 0.97 ± 0.70, and 0.53 ± 0.57 mV (mean ± SD), respectively. It should be noted that responses for some participants were notably small (4 out of 10 subjects for FCR and 1 out of 10 for ECR were <50 μV) during Pre phase, but they were kept in the analysis.

#### 2.3.3. Transcutaneous Spinal Cord Stimulation (tSCS)

Single- (or double-) pulse tSCS can be used to consistently elicit spinal reflexes through the activation of monosynaptic connection between Ia sensory fibers and motoneurons at multiple spinal levels innervating upper-limb muscles [21,23,27]. To evaluate excitability of the spinal reflex circuits in multiple upper-limb muscles simultaneously, a constant current electrical stimulator (DS7A, Digitimer Ltd., Welwyn Garden City, UK) was used to apply a single monophasic square pulse with a 2-ms pulse width [23,27]. As during the conditioning intervention, the cathode electrode (50 × 50 mm) was placed on the spine between C6 and T1 cervical spine processes on the posterior side of the neck (Figure 1), and the anode electrode (75 × 100 mm) was placed along the midline of the anterior side of the neck [23]. Prior to the experiments, the cathode was adjusted to determine the optimal stimulation location. Specifically, tSCS-evoked responses were tested when the cathode was positioned on the C6, C7, or T1 levels with the same stimulus amplitude. The location that induced largest responses in all tested muscles was chosen as the stimulation site (C6: *n* = 0; C7: *n* = 2; T1: *n* = 8). Electrodes were fixed with adhesive tape to prevent their movement during the experiments. Next, to determine the stimulus intensity, the recruitment curves of the responses of all tested upper-limb muscles were obtained for each participant by gradually increasing the tSCS stimulation amplitude. To eliminate the ceiling effect of the evoked responses, the stimulus intensity was adjusted to evoke responses between the lower and middle portions of the ascending part of the recruitment curve in all muscles [21,31] and was kept constant for the duration of the experiment (57.0 ± 4.0 mA). Prior to starting the experiments, a paired-pulse stimulation protocol (50-ms inter-stimulus interval) was applied to confirm whether the evoked responses were initiated in the afferent fibers to evoke PRM reflex responses [23]. Suppression of the second evoked response demonstrated post-activation depression, confirming that PRM reflexes were evoked by activating the afferent roots [23,24,32,33,34,35,36,37]. Notably, the second responses were significantly depressed by the first stimulus activation in all muscles (Section 3.2.1). Specifically, the peak-to-peak amplitudes of the second responses for all recorded muscles were significantly depressed by the first stimulus activation (Wilcoxon signed-rank test, *p* < 0.01; Section 3.2.1), which confirmed the reflex nature of the evoked responses [38].

### 2.4. Conditioning Intervention Using Continuous Cervical tSCS

Continuous cervical tSCS intervention was delivered over the course of 10 min using the same electrode configurations (anode: midline of the anterior side of the neck; cathode: C7 or T1) that were determined for single-pulse tSCS (see Section 2.3.3). Continuous stimulation was applied using a constant current electrical stimulator (Rehab, Chattanooga, DJO Global, Vista, CA, USA) to apply biphasic rectangular pulses with a 400-μs pulse width at a frequency of 30 Hz. Stimulus intensity for each participant was adjusted to evoke paresthesias of the arm muscles by gradually increasing the stimulation pulse amplitude from 0 mA in 1 mA increments until the participants self-reported paresthesias consistent with previous lumbar tSCS study [10,28]. Specifically, participants were asked to identity the abnormal sensation tingling/pricking in the muscles distal to the stimulus delivery. The stimulation amplitudes ranged between 15 and 28 mA, with an average of 20.9 ± 3.4 mA (mean ± SD) between participants. It should be noted that pre assessments were performed just prior to adjusting the stimulating amplitudes to avoid possible effects.

### 2.5. Data Analysis

Peak-to-peak amplitudes were calculated for the TMS-induced MEP responses and tSCS-induced PRM reflex responses of each muscle and for each trial using a custom written script in MATLAB (2017a, The MathWorks Inc., Natick, MA, USA). Eight repeated trials were averaged for each phase (i.e., Pre, During, and Post for MEPs and Pre and Post for PRM reflexes). MEP and PRM reflex amplitudes were then normalized as a percentage of the Pre phase amplitude.

### 2.6. Statistics

Normalized MEP peak-to-peak responses were compared between Pre, During, and Post assessments using the Friedman test, a non-parametric repeated measure one-way analysis of variance (ANOVA). For paired-pulse tSCS protocol, PRM reflex peak-to-peak amplitudes were compared between first and second responses using the Wilcoxon signed-rank test, a non-parametric paired *t*-test. Normalized PRM reflex peak-to-peak responses were compared between Pre and Post assessments using the Wilcoxon signed-rank test. Non-parametric tests were chosen because the Shapiro–Wilk test showed that some identified measures were not normally distributed, and the sample size remains relatively small. Significance level was set to *p* < 0.05. All statistical analyses were performed using SPSS Statistics ver.25 (IBM Corp., Chicago, IL, USA).

## 3. Results

### 3.1. MEP Amplitude

**Figure 2:**
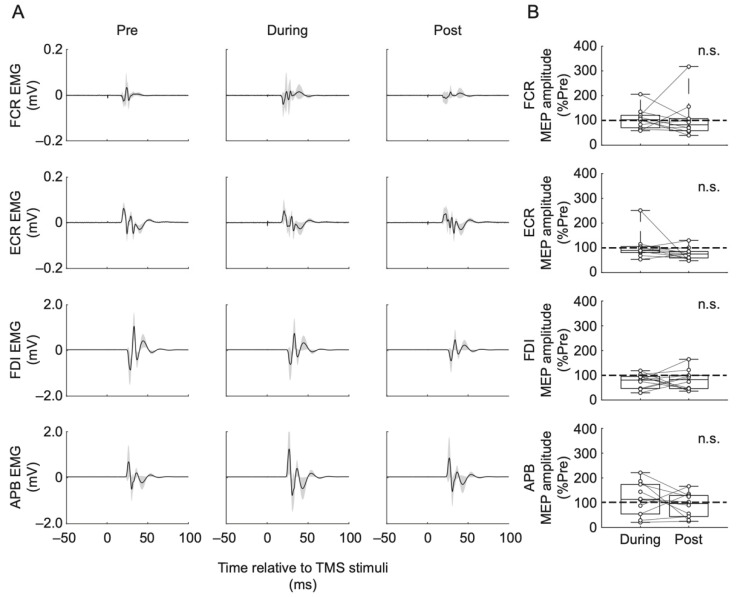
(**A**) Averaged motor evoked potentials (MEPs) for one representative participant at before (Pre), during continuous cervical tSCS (During), and after (Post) assessment points. Shadows represent standard deviation. (**B**) Box plots show MEP amplitude group data in each assessment point. MEP amplitudes were normalized with respect to the MEP amplitude during the Pre assessment (% of Pre) for each participant. Data are shown for the flexor carpi radialis (FCR), extensor carpi radialis (ECR), first dorsal interosseous (FDI), and abductor pollicis brevis (APB) muscles. The horizontal lines in the box plots indicate median values. The ends of the boxes represent the 25th and 75th percentiles. The whiskers on the box plots illustrate the minimum and maximum values. Legend: n.s., non-significant.

The results of the MEP responses are shown in Figure 2. Averaged waveforms obtained from one representative subject are shown in Figure 2A. Box plots indicate the peak-to-peak amplitudes (% Pre) of MEP responses in During and Post phase (Figure 2B). The Friedman test showed that MEP peak-to-peak amplitudes were not statistically significantly different in any of the recorded muscles (FCR: χ^2^(2) = 1.38, *p* = 0.500; ECR: χ^2^(2) = 4.20, *p* = 0.122; FDI: χ^2^(2) = 5.60, *p* = 0.061; APB: χ^2^(2) = 0.60, *p* = 0.741). We also confirmed that comparison of area under the curve of MEP responses in each assessment point were consistent with the results of peak-to-peak amplitudes.

### 3.2. PRM Reflex

#### 3.2.1. Paired-Pulse Protocol

**Figure 3:**
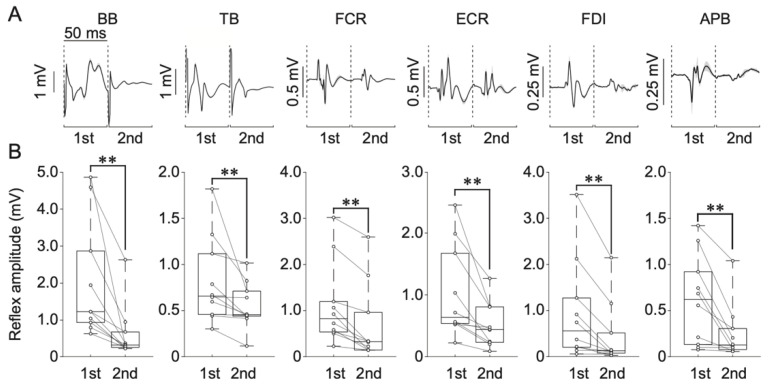
(**A**) Averaged spinal reflex responses for one representative participant during the paired-pulse stimulation protocol. First and second responses were separated by 50 ms. Shadows represent standard deviation. (**B**) Group data for the first and second responses. Data represent the biceps brachii (BB), triceps brachii (TB), flexor carpi radialis (FCR), extensor carpi radialis (ECR), first dorsal interosseous (FDI), and abductor pollicis brevis (APB) upper-limb muscles. The horizontal lines in the box plots indicate median values. The ends of the boxes represent the 25th and 75th percentiles. The whiskers on the box plots illustrate the minimum and maximum values. Legend: ** *p* < 0.01.

The results of the paired-pulse protocol are shown in Figure 3. Averaged waveforms obtained from one representative participant are shown in Figure 3A. Box plots indicate the peak-to-peak amplitude (mV) of first and second responses during the paired-pulse stimulation protocol (Figure 3B). The Wilcoxon signed-rank test showed that the second responses were significantly smaller than first responses in all recorded muscles (BB: Z = 2.803, *p* = 0.005; TB: Z = 2.803, *p* = 0.005; FCR: 2.803, *p* = 0.005; ECR: Z = 2.803, *p* = 0.005; FDI: Z = 2.701, *p* = 0.007; APB: Z = 2.803, *p* = 0.005).

#### 3.2.2. PRM Reflex Amplitude

**Figure 4:**
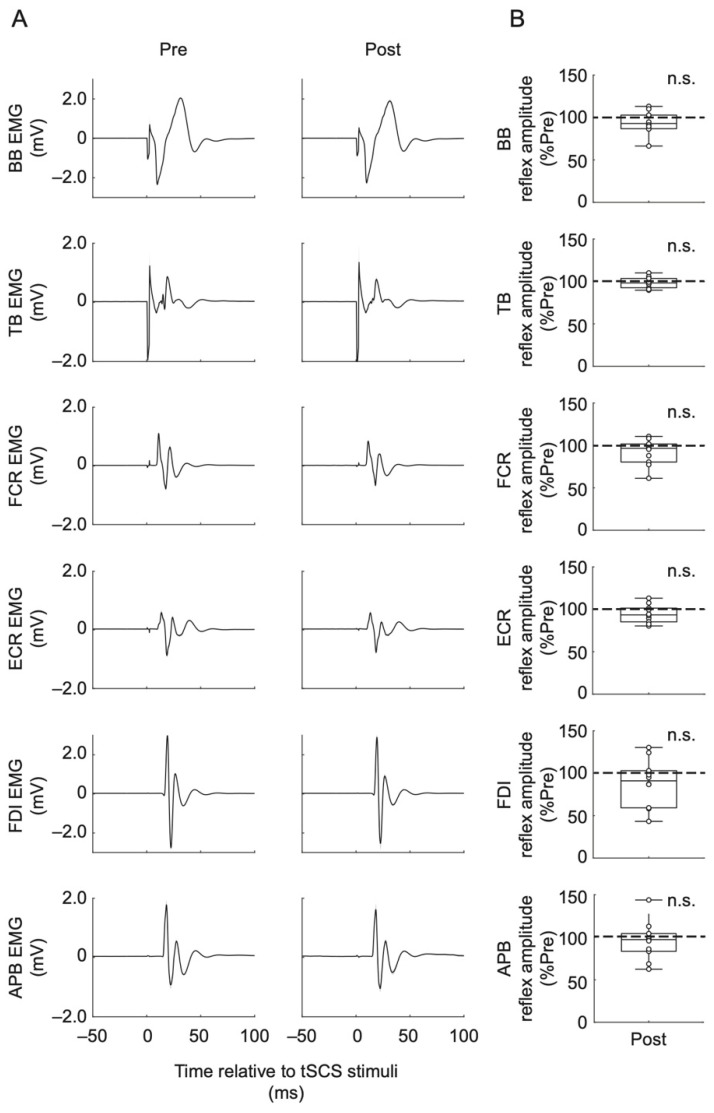
(**A**) Averaged spinal reflex responses for one representative participant at before (Pre) and after (Post) assessment points. Shadows represent standard deviation. (**B**) Box plots show posterior root muscle (PRM) reflex group data in the Post assessment point. PRM reflex amplitudes were normalized with respect to the PRM reflex amplitude during the Pre assessment (% of Pre) for each participant. Data are shown for the biceps brachii (BB), triceps brachii (TB), flexor carpi radialis (FCR), extensor carpi radialis (ECR), first dorsal interosseous (FDI), and abductor pollicis brevis (APB) muscles. The horizontal lines in the box plots indicate median values. The ends of the boxes represent the 25th and 75th percentiles. The whiskers on the box plots illustrate the minimum and maximum values. Legend: n.s., non-significant.

The results of the PRM reflex responses are shown in Figure 4. Average waveforms obtained from one representative participant are shown in Figure 4A. Box plots indicate the peak-to-peak amplitudes (% of Pre) of spinal reflex responses during Post phase (Figure 4B). The Wilcoxon signed-rank test showed that the spinal reflex peak-to-peak amplitudes during Post phase were not statistically significantly different from Pre phase in all recorded muscles (BB: Z = 0.889, *p* = 0.374; TB: Z = 0.415, *p* = 0.678; FCR: Z = 1.01, *p* = 0.314; ECR: Z = 1.01, *p* = 0.314; FDI: Z = 1.24, *p* = 0.214; APB: Z = 0.533, *p* = 0.564).

## 4. Discussion

In the present study, we investigated the effects of relatively short-duration (10 min) and low-intensity (sub-motor-threshold) continuous cervical tSCS applied at rest on the upper-limb muscle MEPs elicited by TMS as well as PRM reflexes elicited by single-pulse cervical tSCS. Our results showed that the intervention did not significantly affect corticospinal (Figure 2) and spinal reflex excitabilities (Figure 4), suggesting that the utilized intervention did not effectively modulate the excitability of spinal monosynaptic connections between Ia sensory afferents and motoneurons and corticospinal pathways in able-bodied participants.

### 4.1. Continuous Cervical tSCS Parameters for Inducing Neuromodulatory Effect

In this study, continuous cervical tSCS was delivered at 30 Hz with biphasic rectangular pulses of 400-μs width and stimulation intensities at a level to elicit paresthesias in the upper-limb muscles for a total duration of 10 min while participants remained in the supine position at rest. Many previous studies reporting therapeutic effects of tSCS used stimulation frequencies between 15 and 50 Hz [6,10,11,12,15,26,28,39,40], while we used 30 Hz in the current study. We also used 400-μs pulse width, which was within the range previously utilized in neuromodulation applications. Specifically, 100-μs pulses were delivered as 10-kHz carrier frequency, within 1-ms bursts at 30 Hz [16,17,18,19]. Additionally, 200 μs pulses were delivered as single pulses or as a train of pulses using a 5-kHz carrier frequency in a recent study by Benavides et al. [15]. Notably, Benavides et al. [15] demonstrated that 200-μs biphasic pulses delivered at 30 Hz can increase corticospinal and spinal excitability in both SCI patients and able-bodied participants without the use of a 5-kHz carrier frequency. While the carrier frequency was reported as beneficial for suppressing pain [41,42], which may reduce discomfort during tSCS [43,44], the choice of stimulating frequency parameters used herein (i.e., 30 Hz without the carrier frequency) is unlikely to be related to the lack of neuromodulation. Moreover, the biphasic rectangular pulse waveform adopted in the present study is also in accordance with other previous neuromodulatory tSCS applications [10,11,16,17,18,19,28]. Additionally, the sub-motor-threshold stimulation intensity applied to elicit paresthesias is consistent with the considerations adopted by Hofstoetter et al. [10,28] for lumbar tSCS interventions in SCI patients. Although the overall stimulation configurations used in the present study are consistent with previous interventions that demonstrated neuromodulation effects in the corticospinal and spinal levels in able-bodied and SCI participants, our intervention did not induce any effects on the studied outcomes, contrary to our hypothesis.

While it may not be possible to attribute a single-stimulation parameter setting to the lack of effects observed herein, next, we discuss the tSCS parameter settings in our study. In particular, our stimulation intervention is generally consistent with the study by Benavides et al. [15], while we adopted longer pulse-width stimulating pulses, lower stimulation amplitude intensities (sub-motor-threshold), and shorter intervention duration. Specifically, by using a twice-longer pulse width (i.e., 400 μs vs. 200 μs used by Benavides et al. [15]) and twice-shorter intervention duration (i.e., 10 min vs. 20 min used by Benavides et al. [15]), the corticospinal and spinal excitability neuromodulation effects were not demonstrated in our study. It is possible that the intervention duration might have influenced our results. Specifically, it was previously demonstrated that 4-min interventions proposed by Parhizi et al. [45] also did not produce neuromodulatory effects. This suggests that the intervention duration may be important when delivering cervical tSCS in the rest conditions. Moreover, it was previously shown that spinal reflex excitability can be modulated using sub-motor-threshold stimulation amplitudes when the intervention was delivered over longer durations, i.e., 30 min for lumbar tSCS [28]. Therefore, while the pulse width and intervention duration trade-off normalized the conditions between our current study and that of Benavides et al. [15], the lower stimulation amplitude (sub-motor-threshold vs. motor-threshold intensity used by Benavides et al. [15]) may suggest that the energy delivery yielded lower efficacy in our current intervention [46]. Taken together, a compensation between stimulation amplitudes and intervention duration during continuous cervical tSCS intervention protocol may have influenced our current results.

It should also be noted that FDI muscle MEPs (target muscle for TMS assessments) during the intervention showed an inhibitory trend (8 of 10 participants had reduced MEP responses during the intervention compared to Pre, as shown in Figure 2B), but there were no statistically significant effects (*p* = 0.061). Additionally, it should also be pointed out that despite the changes in F-wave amplitude and persistency, which indicated change in excitability of cervical spinal circuits in a study by Kumru et al. [19], PRM reflex responses to single-pulse tSCS were not affected after the intervention in their study. On the other hand, cervical tSCS applied at supra-threshold but not at sub-threshold intensities produced lower-limb spinal (H-reflex) facilitation [47]. In addition to the intervention duration, this also points out that intensity of cervical tSCS intervention can be among the important factors underlying spinal reflex excitability. Therefore, future studies are warranted to compare different stimulation amplitude intensities and pulse widths as well as different intervention duration on the neuromodulatory effectiveness.

### 4.2. Voluntary Involvement Combined with Continuous Cervical tSCS May Be Required for Effective Neuromodulation

In addition to the stimulation parameters, voluntary engagement during cervical tSCS may be important to induce neuromodulatory effects. In our current study, continuous cervical tSCS intervention was applied when participants remained at rest in the supine position. On the other hand, many previous studies reporting therapeutic effect of continuous cervical tSCS on upper-limb motor function have delivered stimulation combined with functional task performance [16,17,18,26]. Voluntary engagement combined with afferent recruitment may elicit a form of Hebbian plasticity in the CNS during application of electrical stimulation [48,49]. A recent study reported that continuous cervical tSCS combined with hand training enhanced hand motor outputs, increased F-wave amplitudes and persistency, reduced TMS-induced resting motor thresholds, and facilitated MEP amplitudes after the intervention [19]. In contrast, cervical tSCS without hand training only increased the F-wave amplitudes [19]. Therefore, a neuromodulatory effect resulting from short-duration or single-session continuous cervical tSCS may depend on the brain state enhanced by voluntary drive when stimulation is delivered. Taken together, voluntary engagement may be essential to maximize neuromodulatory effects of cervical tSCS during short-term interventions.

### 4.3. Electrode Configuration Considerations for Cervical tSCS

In our current study, the cathode electrode was placed on the spine between the C7 and T1 cervical spinal processes on the posterior side of the neck, and the anode electrode was placed along the midline of the anterior side of the neck (Figure 1) [21,23,27]. Previous studies investigating the neuromodulatory effects of cervical tSCS placed anode electrodes bilaterally over the iliac crests [15,16,19]. Recent findings revealed that anode configuration over the anterior neck may elicit larger spinal reflex responses compared to when anode electrode were placed over iliac crests bilaterally, while there were no differences in the discomfort experienced between these different configurations at similar stimulation intensities [21]. Although it is also plausible that the anode location can affect the effectiveness of cervical tSCS intervention, it is unlikely that it was a critical factor because our previous work demonstrated that the anode placement over the anterior neck effectively targets the spinal circuits [21]. Future studies should also consider optimizing the placement of the anode electrode during continuous cervical tSCS so as to minimize discomfort.

### 4.4. Limitations

Our work has several limitations that should be noted. First, although neurophysiological effect of stimulation parameters used in our current study (i.e., sub-threshold intensity continuous tSCS) has not been investigated in previous cervical tSCS applications, the parameters used herein are not completely novel since similar approaches have been used with lumbar tSCS [28]. Therefore, future studies should explore more optimal stimulation settings for cervical tSCS. Second, we did not investigate neurophysiological effects in patients with neurological injury, such as spinal cord injury. For the clinical application of cervical tSCS, neurophysiological effect in patients should also be investigated using various stimulation parameters in the future. Third, we assessed corticospinal excitability using TMS during delivery of cervical tSCS, while spinal reflex excitability could not be assessed during cervical tSCS application since same electrodes were used for eliciting PRM reflex responses and for delivering the intervention. Therefore, spinal excitability during delivery of tSCS should be investigated using other assessment methods, such as H-reflex or F-wave, although it is well known that these methods can be applied to assess only a limited number of upper-limb muscles [50]. Finally, our current study was not able to identify the minimum time required for inducing neurophysiological effectiveness of cervical tSCS. This is very important for optimizing cervical tSCS stimulation-parameter settings. Therefore, future studies should consider the dose-response relationship to identify a minimum time required for effective application of cervical tSCS.

## 5. Conclusions

We investigated effects of continuous cervical tSCS on corticospinal and spinal reflex excitability. Our results showed that low-intensity and short-duration continuous cervical tSCS intervention applied at rest did not significantly affect corticospinal and spinal reflex excitability in able-bodied subjects. In addition to the amplitude and the duration of the intervention; the stimulating pulse and/or the pulse width, including the carrier frequency of the stimulating pulses; as well as voluntary engagement during the intervention may be important for inducing short-term neuromodulatory effect of cervical tSCS. Therefore, future studies should consider stimulation-parameters settings while minimizing stimulation-induced discomforts during cervical tSCS in actual clinical applications.

## Data Availability

The data related to the findings of this study are available from the corresponding author, upon reasonable request.
